# Supplementary material for: Optimal searching behaviour generated intrinsically by the central pattern generator for locomotion
Source: eLife. 2019 Nov 1;8:e50316. doi: 10.7554/eLife.50316 (PMC6879304; doi:10.7554/eLife.50316)
Supplement: Supplementary file 2. — (A) Kalman filter (KF) parameters and (B) minimum step resolution values were altered to determine the effects of such changes on the consistency of treatment µ values. The values used for the results presented in the main paper were Position and Velocity minimum variances of 0.5 and a covariance of 1.0 for the KF, and a minimum step resolution of 0.44. The sensitivity analysis for the KF parameters considered values that differed significantly from those used, bracketing the analysis values. As can be seen in (A), differences in the µ values were generally small, confirming that the values chosen for the KF did not alter the finding of truncated power-laws in larva tracks. Rather, the average µ values from all KF sensitivity tests are close to those found in the analysis. The minimum step resolution value chosen for the analysis (0.44) was determined from the tracking resolution and larval movements (head sways and peristaltic contractions) and represents the lowest value above the track noise. All computed move steps lower than this value were excluded from the analysis. For the sensitivity test, values of 0.3, 0.5, 0.7 and 0.9 were used as this range covered viable alternative values. As with the KF tests, the finding of truncated power-laws and the resultant µ values differed very little from those presented in the original analysis. We conclude that significant changes in parameters associated with video track processing had no important effects on our finding of truncated power-laws in larva movement paths and the resultant µ values. [file elife-50316-supp2.docx]

**Supplementary File 2.** Sensitivity analyses for Kalman filter parameters and minimum step resolution values.

(A)

|  |  | Kalman filter analysis µ values | | | | | | |
| --- | --- | --- | --- | --- | --- | --- | --- | --- |
|  |  | Position & velocity | | | Covariance | | |  |
| Treatment | mu | 1 | 0.1 | 0.05 | 2 | 0.5 | 0.1 | Average |
| BL/+ 33 Deg | 1.45 | 1.48 | 1.38 | 1.42 | 1.40 | 1.48 | 1.45 | 1.44 |
| BLsens-shi | 2.29 | 2.28 | 2.30 | 2.28 | 2.30 | 2.28 | 2.19 | 2.27 |
| BL-shi | 2.13 | 2.09 | 2.07 | 2.08 | 2.12 | 2.08 | 2.13 | 2.10 |
| BL/+ 22 Deg | 1.62 | 1.77 | 1.61 | 1.61 | 1.64 | 1.77 | 1.79 | 1.70 |
| MB247/+ | 2.22 | 2.38 | 2.20 | 2.10 | 2.20 | 2.38 | 2.27 | 2.26 |
| MB247-shi | 1.17 | 1.24 | 1.16 | 1.15 | 1.16 | 1.24 | 1.31 | 1.21 |
| BLsens-rprhid | 1.98 | 1.99 | 2.03 | 2.05 | 1.98 | 2.00 | 2.01 | 2.01 |
| BLsens-rprhid_control | 1.55 | 1.48 | 1.48 | 1.43 | 1.50 | 1.57 | 1.58 | 1.51 |
| shi/+ 22 Deg | 1.31 | 1.43 | 1.21 | 1.24 | 1.27 | 1.43 | 1.40 | 1.33 |
| shi/+ 33 Deg | 1.35 | 1.36 | 1.56 | 1.16 | 1.71 | 1.36 | 2.31 | 1.58 |

(B)

|  |  | Minimum step analysis µ values | | | | | |
| --- | --- | --- | --- | --- | --- | --- | --- |
|  |  | Minimum step resolution (mm) | | | |  |  |
| Treatment | mu | 0.3 | 0.5 | 0.7 | 0.9 | Average | Overall average |
| BL/+ 33 Deg | 1.45 | 1.46 | 1.46 | 1.44 | 1.42 | 1.45 | 1.44 |
| BLsens-shi | 2.29 | 2.25 | 2.26 | 2.14 | 3.90 | 2.64 | 2.42 |
| BL-shi | 2.13 | 2.13 | 2.13 | 2.13 | 2.13 | 2.13 | 2.11 |
| BL/+ 22 Deg | 1.62 | 1.67 | 1.64 | 1.60 | 1.59 | 1.63 | 1.67 |
| MB247/+ | 2.22 | 2.12 | 2.24 | 2.29 | 2.02 | 2.17 | 2.22 |
| MB247-shi | 1.17 | 1.20 | 1.16 | 1.16 | 1.22 | 1.19 | 1.20 |
| BLsens-rprhid | 1.98 | 1.98 | 2.01 | 1.99 | 1.95 | 1.98 | 2.00 |
| BLsens-rprhid_control | 1.55 | 1.58 | 1.51 | 1.48 | 1.67 | 1.56 | 1.53 |
| shi/+ 22 Deg | 1.31 | 1.40 | 1.21 | 1.20 | 1.23 | 1.26 | 1.30 |
| shi/+ 33 Deg | 1.35 | 1.29 | 1.25 | 1.25 | 1.60 | 1.35 | 1.49 |
