# Supplementary material for: Optimal searching behaviour generated intrinsically by the central pattern generator for locomotion
Source: eLife. 2019 Nov 1;8:e50316. doi: 10.7554/eLife.50316 (PMC6879304; doi:10.7554/eLife.50316)
Supplement: Supplementary file 5. — Larva tracks with fitted move steps were separated at the midpoint and those for which each half was best fitted by a truncated power-law were retained for analysis. The average µ values for the first and second half of all tracks across trials within a treatment were compared to determine any significant differences, which would indicate changes in track statistics over time (i.e. non-stationarity). We found no significant differences between the first and second halves of the tracks and with no clear trend of increasing or decreasing µ values, as might be expected to occur if µ showed significant temporal dependence on changing satiety or other factors over the 1 hr trial period. [file elife-50316-supp5.docx]

**Supplementary File 5.** Tests for stationarity in the larva movement pattern data within treatments.

| Treatment | First half | n | Second half | n | p-value | test |
| --- | --- | --- | --- | --- | --- | --- |
| BL/+ 22 Deg | 1.47 | 21 | 1.41 | 22 | 0.932 | Rank sum |
| BL/+ 33 Deg | 1.37 | 18 | 1.42 | 20 | 0.759 | Rank sum |
| BLsens-shi | 1.84 | 7 | 2.16 | 5 | 0.106 | Rank sum |
| BL-shi | 1.77 | 16 | 1.56 | 13 | 0.228 | Rank sum |
| MB247/+ | 1.63 | 19 | 1.51 | 15 | 0.375 | t |
| MB247-shi | 1.58 | 16 | 1.58 | 19 | 0.882 | Rank sum |
| BLsens-rprhid | 2.10 | 14 | 2.17 | 15 | 0.710 | t |
| BLsens-rprhid_control | 1.41 | 45 | 1.47 | 53 | 0.148 | Rank sum |
| shi/+ 22 Deg | 1.46 | 25 | 1.57 | 24 | 0.342 | Rank sum |
| shi/+ 33 Deg | 1.50 | 32 | 1.45 | 31 | 0.268 | Rank sum |
